# Clinical characteristics and outcomes of hospitalized patients with SARS-CoV-2 infection in a Latin American country: Results from the ECCOVID multicenter prospective study

**DOI:** 10.1371/journal.pone.0258260

**Published:** 2021-10-08

**Authors:** Ezequiel Cordova, Analia Mykietiuk, Omar Sued, Lautaro De Vedia, Natalia Pacifico, Matias H. Garcia Hernandez, Natalia M. Baeza, Franco Garibaldi, Maria Fernanda Alzogaray, Rosa Contreras, Lucrecia Soler Puy, Pablo G. Scapellato, Laura Barcelona, Mariana L. Golikow, Maria Florencia Piñeiro, Hugo J. Miño, Maria Fernanda Consalvo, Corina Nemirovsky, Marisa Sanchez, Myrna Cabral, Lucia Lamponi Tappata, Mariano Blasco, Jamile Ballivian, Gustavo Lopardo, Martin E. Stryjewski, on behalf of the ECCOVID study group

**Affiliations:** 1 Hospital Cosme Argerich, Ciudad Autónoma de Buenos Aires (CABA), Buenos Aires, Argentina; 2 Instituto Medico Platense—La Plata, Buenos Aires, Argentina; 3 Fundación Huésped—CABA, Buenos Aires, Argentina; 4 Hospital F.J. Muñiz—CABA, Buenos Aires, Argentina; 5 Centro de Educación Médica e Investigaciones Clínicas (CEMIC)—CABA, Buenos Aires, Argentina; 6 Hospital Dr. Marcial V. Quiroga—San Juan, San Juan, Buenos Aires, Argentina; 7 Hospital Julio C. Perrando—Resistencia, Chaco, Buenos Aires, Argentina; 8 Hospital Santojanni—CABA, Buenos Aires, Argentina; 9 Hospital Prof. Dr. Bernardo A. Houssay—Vicente Lopez, Buenos Aires, Argentina; 10 Hospital Nacional Posadas—El Palomar, Buenos Aires, Argentina; 11 Hospital Fernandez—CABA, Buenos Aires, Argentina; 12 Hospital Iturraspe—Santa Fe, Santa Fe, Argentina; 13 Hospital Penna–CABA, Buenos Aires, Argentina; 14 Hospital Italiano—CABA, Buenos Aires, Argentina; 15 Hospital Central—Mendoza, Mendoza, Argentina; 16 Hospital Leonidas Lucero—Bahía Blanca, Buenos Aires, Argentina; 17 Sanatorio Agote—CABA, Buenos Aires, Argentina; 18 Helios Salud–CABA, Buenos Aires, Argentina; Fundacao Oswaldo Cruz, BRAZIL

## Abstract

**Background:**

Clinical features and outcomes of SARS-CoV-2 infections diverge in different countries. The aim of this study was to describe clinical characteristics and outcomes in a cohort of patients hospitalized with SARS-CoV-2 in Argentina.

**Methods:**

Multicenter prospective cohort study of ≥18 years-old patients with confirmed SARS-CoV-2 infection consecutively admitted to 19 hospitals in Argentina. Multivariable logistic regression models were used to identify variables associated with 30-day mortality and admission to intensive care unit (ICU).

**Results:**

A total of 809 patients were analyzed. Median age was 53 years, 56% were males and 71% had at least one comorbidity. The most common comorbidities were hypertension (32%), obesity (23%) and diabetes (17%). Disease severity at admission was classified as mild 25%, moderate 51%, severe 17%, and critical 7%. Almost half of patients (49%) required supplemental oxygen, 18% ICU, and 12% invasive ventilation. Overall, 30-day mortality was 11%. Factors independently associated with ICU admission were male gender (OR 1.81; 95%CI 1.16–2.81), hypertension (OR 3.21; 95%CI 2.08–4.95), obesity (OR 2.38; 95%CI 1.51–3.7), oxygen saturation ≤93% (OR 6.45; 95%CI 4.20–9.92) and lymphopenia (OR 3.21; 95%CI 2.08–4.95). Factors independently associated with 30-day mortality included age ≥60 years-old (OR 2.68; 95% CI 1.63–4.43), oxygen saturation ≤93% (OR 3.19; 95%CI 1.97–5.16) and lymphopenia (OR 2.65; 95%CI 1.64–4.27).

**Conclusions:**

This cohort validates crucial clinical data on patients hospitalized with SARS-CoV-2 in Argentina.

## Introduction

The novel epidemic of severe acute respiratory syndrome coronavirus 2 (SARS-CoV-2) producing COVID-19 disease has rapidly escalated to pandemic proportions [1]. To date over one hundred million humans have been infected and around two million have died due to COVID-19 [2]. Several reports all over the world have described clinical features and outcomes of such patients [3–6]. However, these studies may differ widely in different countries and regions [1, 7]. In addition, many clinical observations from specific cities, states or countries may not apply to other territories or populations. Therefore, it is crucial to have prospective clinical information on the outcomes and predictors on COVID-19 in each country or region.

On March 3rd, 2020, the first case in Argentina was confirmed. Since then, more than two million subjects have been infected and over 50 thousand have died with COVID-19 in the country [2]. Epidemiological studies conducted in Argentina are already available [8, 9]. However, these studies lack detailed clinical information. Therefore, such clinical data is still needed in the country.

The aim of this study was to describe the clinical characteristics, outcomes and predictors of ICU admission and death in a multicenter prospective cohort of patients hospitalized with COVID-19 in Argentina.

## Methods

### Study design

The ECCOVID study is an ongoing multicenter prospective observational cohort study conducted in 19 hospitals in Argentina. The aim of the study is to assess clinical and epidemiological characteristics of patients admitted with COVID-19 in the country. The study was designed by the Sub-committee of Research at the Argentinean Society of Infectious Diseases (SADI)

### Participants

Patients were enrolled in the study if they had ≥18 years old, were admitted in a participating center, had SARS-CoV-2 infection confirmed by polymerases chain reaction (PCR) or other validated methods on nasopharyngeal swab or other respiratory specimens, and had consented participation in the study. Patients were consecutively enrolled between March 3^rd^, 2020, and October 15^th^, 2020.

### Data collection

Routine care data was prospectively extracted from the medical records and entered into a Redcap database (Research Electronic Data Capture, Vanderbilt University, US) hosted by SADI. Main clinical, epidemiological, radiological and laboratory variables were captured within 24 hours from admission, respectively. Patients were followed during hospitalization and at 30, 60 and 180 days. For those patients who were alive and not hospitalized a telephonic follow up was accepted. All relevant treatments and interventions were obtained during hospitalization. Disease severity was classified as mild, moderate, severe, or critical [10].

### Outcomes

The main outcome for this analysis was mortality at 30-days from the index admission. The secondary outcome was admission at the intensive care unit (ICU).

### Missing data

As the main and secondary outcomes of the study were mainly descriptive, no imputation of missing data was performed. To reduce the impact of missing data on outcome analyses, we restricted such analyses to patients who had been admitted for at least a month before data extraction.

### Statistical analyses

Categorical variables were described using absolute and relative frequencies. Continuous variables were described using medians and interquartile ranges (IQR). Comparisons between groups were made using Mann Whitney U test or Kruskal-Wallis test for continuous variables, or *X*^*2*^ *test* for categorical variables, as appropriate. All tests were two-sided and considered significant if p-value was less than 0.05.

To investigate the effect of different factors on 30-day mortality, we performed a cross sectional analysis using a multivariable logistic regression model. Backwards stepwise model selection with a p-value of 0.05 was conducted. However, variables known from previous studies to be associated with 30-day mortality were forced into the model. To assess the fit of the model, we performed Hosmer-Lemeshow goodness of fit test. Data analyses were performed using R (R Core Team version 4.0.3, Vienna, Austria).

### Ethics

Informed consent was obtained according to each participating Ethic Committees.

## Results

From March 3^rd^ to October 15^th^, 2020, 1074 patients with ≥18 years old who had at least 30-day follow up were prospectively enrolled in the ECCOVID study. Critical variables were fully available in 809 of these subjects who were included in the present analysis. The median age was 53 years (IQR 38–67); 56% were males and 71% had at least one comorbidity. The most common comorbidities were hypertension (32%), obesity (23%) and diabetes (17%). Baseline characteristics in the overall population and by gender are shown in **Table 1**.

**Table 1:** Baseline characteristics among 809 hospitalized patients with COVID-19 in Argentina.

Characteristics	Male	Female	All
	n = 456	n = 353	n = 809
Age at admission median (interquartile range) in years	54 (38–70)	52 (38–65)	53 (38–67)
< 60 years	306 (67.1%)	215 (60.9%)	521 (64.4%)
> 60 years	150 (32.9%)	138 (39.1%)	288 (35.6%)
Any comorbidity			
No	140 (30.7%)	93 (26.3%)	233 (28.8%)
Yes	316 (69.3%)	260 (73.7%)	576 (71.2%)
Diabetes			
No	384 (84.2%	287 (81.3%)	671 (82.9%)
Yes	72 (15.8%)	66 (18.7%)	138 (17.1%)
Chronic pulmonary disease, not asthma			
No	438 (96.1%)	344 (97.5%)	782 (96.7%)
Yes	18 (3.9%)	9 (2.5%)	27(3.3%)
Asthma			
No	435 (95.4%)	321 (90.9%)	756 (93.4%)
Yes	21 (4.6%)	32 (9.1%)	53 (6.6%)
Hypertension			
No	323 (70.8%)	228 (64.6%)	551 (68.1%)
Yes	133 (29.2%)	125 (35.4%)	258 (31.9%)
Obesity			
No	355 (77.9%)	266 (75.4%)	621 (76.8%)
Yes	101 (22.1%)	87 (24.6%)	188 (23.2%)
Chronic kidney disease			
No	433 (95%)	343 (97.2%)	776 (95.9%)
Yes	23 (5%)	10 (2.8%)	33 (4.1%)
AIDS/HIV			
No	439 (96.3%)	347 (98.3%)	786 (97.2%)
Yes	17 (3.7%)	6 (1.7%)	23 (2.8%)
Solid malignancy			
No	438 (96.1%)	335 (94.9%)	773 (95.6%)
Yes	18 (3.9%)	18 (5.1%)	36 (4.4%)
Cardiac disease			
No	418 (91.7%)	332 (94.1%)	750 (92.7%)
Yes	38 (8.3%)	21 (5.9%)	59 (7.29%)
Immunosuppression			
No	442(96.9%)	345 (97.7%)	787 (89.2%)
Yes	14 (3.1%)	8 (2.3%)	87 (10.8%)
Death at 30-day follow up			
No	399 (87.5%)	321 (90.93%)	720 (89%)
Yes	57 (12.5%)	32 (9.07%)	89 (11%)

Most frequent symptoms reported prior to or at admission were fever (61%), cough (60%) and dyspnea (40%) **(Table 2).** At admission almost one third of the patients (30%) had abnormal body temperature (≥37.5°C) and 18.5% had fever (≥38°C). Disease severity at admission was mild in 25%, moderate in 51%, severe in 17%, and critical in 7% of patients. Oxygen saturation ≤93% was observed in about 20% cases. The median time from symptoms onset to hospitalization was 5 days (IQR 2–7). Approximately one fifth of the patients (21%) did not present symptoms of upper or lower respiratory tract infection.

**Table 2:** Disease severity, signs, symptoms, and radiology findings among 809 hospitalized patients with COVID-19 in Argentina.

VARIABLE	N (%)
**Disease severity**	
Mild	204 (25%)
Moderate	409 (51%)
Severe	139 (17%)
Critical	57 (7%)
**Signs**	
Temp 37.5–37.9°C (n = 779)	91 (11.6%)
Temp ≥38°C (n = 779)	144 (18.5%)
Respiratory rate ≥20 x min	416 (51.4%)
Heart rate ≥100 x min	218 (26.9%)
Oxygen saturation ≤ 93%	172 (21.3%)
**Symptoms**	
Cough	489 (60.4%)
Shortness of breath	327 (40.4%)
Myalgia	238 (29.4%)
Sore throat	234 (28.9%)
Headache	149 (18.4%)
Anosmia	148 (18.3%)
Ageusia	129 (15.9%)
Diarrhea	108 (13.3%)
Chilling	97 (12.0%)
Expectoration	64 (7.9%)
Chest pain	59 (7.3%)
Rhinorrhea	55 (6.8%)
Abdominal pain	46 (5.7%)
Conjunctival congestion	9 (1.1%)
Other	55 (6.8%)
**Radiology findings**	
**Chest radiography (n = 618)**	
Abnormal findings	365 (59.5%)
Bilateral interstitial infiltrate	277 (75.9%)
Unilateral interstitial infiltrate	56 (15.3%)
Unilateral consolidation pattern	56 (15.3%)
Bilateral consolidation pattern	35 (9.6%)
Pleural effusion	10 (2.7%)
**Chest computer tomography (n = 397)**	
Abnormal findings	340 (85.6%)
Bilateral ground glass opacities	291 (85.6%)
bilateral consolidation	60 (17.6%)
Unilateral ground glass opacities	34 (10%)
Unilateral consolidation	29 (8.5%)
Pleural effusion	19 (5.6%)

Chest Radiographs were performed at admission in 618 patients (76%). Abnormal findings were present in almost 60% of these patients. The most common radiographic patterns were bilateral interstitial infiltrates (75.9%). Chest computed tomography (CT) was obtained in 397 patients (49%). Abnormal findings were present in 86% of such patients. Bilateral ground glass opacities were the most frequent finding on CT. Over 20% of the patients with normal chest radiograph had lung infiltrates on the chest CT. In patients who were admitted without respiratory symptoms, radiographic infiltrates on chest radiographs or CT were observed in 38.6% and 72.2% of cases, respectively. Laboratory findings at baseline are presented in **Table 3**. Lymphopenia was observed in 31% of the patients. Elevated alanine transaminase (ALT) or aspartate transaminase (AST) (2 times above the upper normal limit) was found in 15.9% of the patients. D-dimer levels were available in 420 patients and found to be ≥1.0 μg/mL in 25% of these patients. Patients with severe or critical COVID-19 had lower lymphocyte count (1100 vs 1400 cells/mL; p<0.001), higher C-reactive protein (CRP) (27.5 mg/L vs. 6 p<0.001), D-dimer (0.9 vs. 0.5 μg/ml; p<0.001) and ferritin levels (780 vs. 344 ng/mL; p<0.001), respectively.

**Table 3:** Laboratory findings at hospital admission among patients with COVID-19 in Argentina.

Variable	N	Median (IQR) or %
Hemoglobin (g/dL)	799	13.6 (12.2–14.7)
White blood cell count (x10^9^ /L)	809	6.4 (4.87–8.5)
Lymphocytes (x10^9^ /L)	809	1.3(0.9–1.8)
≤ 1 x x10^9^ /L	252	31.1%
Platelets (x10^9^ /L)	750	220 (176–277)
≤ 150 x 10^9^ /L	750	10.9%
Creatinine (mg/dL)	791	0.80 (0.66–0.98)
≥ 1.5	50	6.3%
AST (U/L)	773	32.5 (23–53)
ALT (U/L)	773	32 (19–56)
Increased AST and/or ALT ≥ x2 UNL	123	15.9%
Lactate dehydrogenase (LDH) (U/L)	653	442 (327–617)
≥500	272	41.7%
C-reactive protein (mg/L)	420	10 (2.7–35)
≥ 1	360	85.5%
D-dimer (μg/ml)	420	0.53 (0.30–1)
≥1	107	25.5%
Ferritin (ng/mL)	391	415 (158–922)
>500	172	44%

ALT: alanine transaminase; AST: aspartate transaminase; UNL: upper normal limit.

During hospitalization almost half (49%) required oxygen supplementation, 18% ICU, and 12% invasive mechanical ventilation, respectively. Of those admitted to ICU, 70% required mechanical ventilation. The median time to ICU admission was 7 days (IQR 4–9) from onset of symptoms and 1 day (IQR 0–4) from hospitalization. The median duration of mechanical ventilation was 12 days (IQR 6–18).

**Fig 1:**
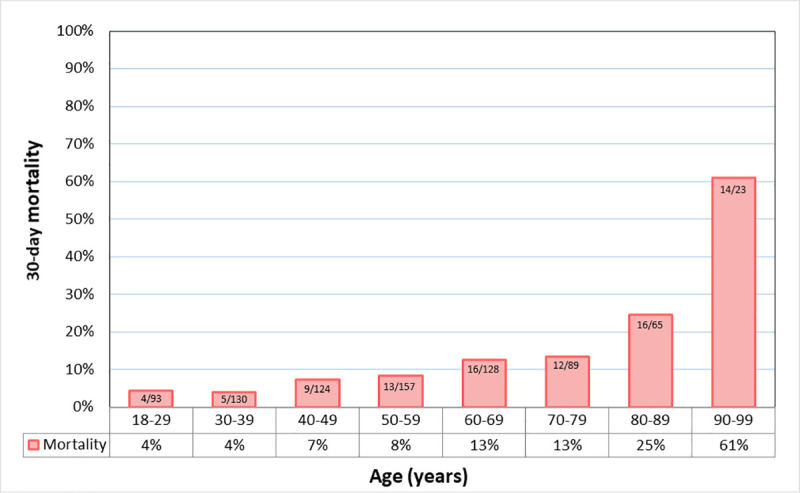
30-day mortality by age group among 809 patients hospitalized with COVID-19 in Argentina.

Overall, 30-day mortality was 11%. The median time between hospital admission and death was 15 days (IQR 9–21). In patients requiring oxygen supplementation mortality at 30 days was 10.8% for patients in the general ward, 36.6% in those admitted to ICU, and 50.5% in patients undergoing invasive mechanical ventilation. Clinical outcomes are displayed in **Table 4** whereas Fig **1** shows 30-day mortality by age group.

**Table 4:** Clinical outcomes and dispositions among 809 patients hospitalized with COVID-19 in Argentina.

Variable	N	Median (IQR) or (%)
Admission at general ward	752	93%
Admission at ICU	57	7%
Step up ICU admission ^a^	85	11.3%
Oxygen requirement	392	48.5%
Mechanical ventilation	97	12%
Duration of mechanical ventilation (days)	97	12 (6–18)
Shock	88	10.9%
Renal replacement therapy	17	2.1%
Length of stay in general ward (days) ^b^	667	10 (6–15)
Length of stay in ICU (days)^c^	142	10 (5–19)
Do-not-resuscitate status	39	4.8%
Discharged at 30 days	665	82.2%
Death at 30-days	89	11%
Among patients in ICU	52	36.6%

ICU: intensive care unit.

a. Among patients initially admitted to a general ward (n = 752).

b. From admission to discharge or death.

c. From ICU admission to discharge to a general ward or death.

In the multivariable analysis factors independently associated with ICU admission were male gender (OR 1.81; 95% CI 1.16–2.81), arterial hypertension (OR 3.21; 95% CI 2.08–4.95), obesity (OR 2.38; 95%CI 1.51–3.7), oxygen saturation ≤93% (OR 6.45; 95% CI 4.20–9.92) and lymphopenia (lymphocyte count <1000 cell/mL) (OR 3.21; 95%CI 2.08–4.95) (Hosmer-Lemeshow X^2^ = 8.4, df = 7, p-value = 0.30) (**Table 5).** Factors at admission independently associated with 30-day mortality in the multivariable model included age ≥60 years-old (OR 2.68; 95% CI 1.63–4.43), oxygen saturation ≤93% at admission (OR 3.19; 95%CI 1.97–5.16) and lymphopenia (OR 2.65; 95% CI 1.64–4.27) (Hosmer-Lemeshow X^2^ = 3.36, df = 8, p-value = 0.91) **(Table 6).**

**Table 5:** Multivariable analysis of characteristics associated with intensive care unit admission among 809 patients with COVID -19 in Argentina.

Variables	Unadjusted OR	Adjusted OR
	(95% CI)	(95% CI)
Gender male	1.75 (1.19–2.58)	1.81 (1.16–2.81)
Age ≥ 60 years	1.49 (1.03–2.17)	0.90 (0.54–1.53)
Oxygen saturation ≤93% at admission	7.65 (5.13–11.42)	6.45 (4.20–9.92)
Lymphocyte count <1000 cell/ml at admission	3.32 (2.28–4.84)	3.21 (2.08–4.95)
Arterial hypertension	2.21 (1.66–4.53)	1.93 (1.14–3.26)
Obesity	2.68 (1.81–3.96)	2.38 (1.51–3.7)
COPD	0.60 (0.18–2.02)	0.32 (0.08–1.21)
Diabetes	1.97 (1.28–3.04)	0.91 (0.53–1.59)
Cardiac disease	1.57 (0.84–2.95)	0.96 (0.45–2.1)
Solid malignancy	0.43 (0.13–1.42)	0.44 (0.12–1.61)

**Table 6:** Multivariable analysis of characteristics associated with death at 30-days among 809 patients hospitalized with COVID-19 in Argentina.

Variables	Unadjusted OR	Adjusted OR
	(95% CI)	(95% CI)
Gender male	1.43 (.91–2.26)	1.43 (.87–2.33)
Age ≥ 60 years	3.77 (2.38–5.97)	2.68 (1.63–4.43)
Oxygen saturation ≤93% at admission	4.28 (2.71–6.76)	3.19 (1.97–5.16)
Lymphocyte count <1000 cell/ml at admission	3.65 (2.33–5.74)	2.65 (1.64–4.27)
Any comorbidity	3.16 (1.65–6.06)	1.76 (0.87–3.55)

## Discussion

In this prospective multicenter study, we analyzed over 800 patients hospitalized with COVID-19 in Argentina. To our knowledge this is the first large and detailed clinical report in our country which provides several important findings allowing better understanding on the pandemic in the country.

First, the current study comprehensively describes clinical and demographic characteristics of patients admitted with SARS-Cov-2 infection in Argentina. Overall, we found in our study population similar comorbidities to those prevalent in different COVID-19 reports such as hypertension, obesity, and diabetes [4–6]. The median age in our cohort of patients (52 years old) was comparable to those reported in large epidemiological studies including hospitalized patients with COVID-19 in Argentina [8] and elsewhere [5, 6]. The most frequent symptoms among hospitalized patients with COVID-19 were fever, cough, and shortness of breath. Anosmia and ageusia were found in less than 20% of cases which were also in agreement with other published cohorts [5, 6, 8, 9, 11, 12]. Interestingly only 20% of our patients had fever (≥ 38C) and a similar percentage lacked any respiratory symptoms at admission. Taken together this information indicates that neither fever nor respiratory symptoms are reliable markers of infection due to SARS-CoV-2 among patients requiring hospitalization.

Second, both radiologic and laboratory findings were also important. Most patients in our study had evidence of lung infiltrates on chest images at admission. As previously reported, chest CT had a better diagnostic accuracy than chest radiographic [13]. Characteristics of the lung infiltrates were similar to other descriptions [5, 14, 15]. In addition, we were also able to identify infiltrates in a significant proportion of those individuals without respiratory symptoms (72.2%). A CT scan is a valuable diagnostic tool that help to diagnose COVID-19 in patients with either difficult or negative images on the chest X rays [14, 16] as well as in those patients with absence of respiratory symptoms. In the laboratory analysis we have also found markers of systemic inflammation such as CRP in a vast majority of our patients (in whom the test was performed). The current knowledge indicates that patients with severe COVID-19 or those with adverse outcomes have increased levels of inflammatory cytokines as well as other infection-related biomarkers [12, 17–20]. However, these laboratory parameters, mainly D-dimer, CRP and ferritin are not easily available in health-care centers with limited resources. In the present cohort, only around half of our patients had CRP or D-dimer determined at admission. Therefore, simple laboratory parameters should be used to identify patients at risk of adverse outcomes in our population.

Third, the outcomes of our patients are also worthy to mention. In our study the median time from symptoms onset to hospitalization or ICU admission was 5 and 7 days, respectively. These findings are consistent with other reports [18, 21, 22]. Similarly, the overall 30-day mortality in our cohort of patients was 11%. Solidarity trial with a total of 11,330 patients from 405 hospitals in 30 countries reported a 28-day in-hospital death of 11.8% [23]. Near half of patients in our study requiring mechanical invasive ventilation died. This mortality rate also reflects the one reported in a recent meta-analysis with over 55.000 patients in 69 studies across 23 countries [24].

Finally, risk factors for ICU admission were male sex, arterial hypertension, obesity, oxygen saturation ≤93% and lymphopenia at admission. These risk factors identified are in agreement with other investigations [22, 25]. Age over 60 years was not found as an independent risk factor for ICU admission in our study. However, we found that this variable was associated with 30-day mortality. A possible explanation for these contradictory results is that older patients (≥80 year) were not necessarily transferred to ICUs based on several medical, familiar, or legal considerations [26]. On the other hand, independent risk factors for 30-day mortality sorted out by our study were age over 60 years old, oxygen saturation ≤93% and lymphopenia. In contrast to other reports [6, 9, 27], we did not detect an independent effect of gender or comorbidities on 30-day mortality. Probably, these variables were attenuated by the strong effect of age, markers of severe lung involvement such as hypoxemia and/or immune alterations such as lymphopenia.

Our study had some limitations. First, the adverse clinical outcomes may be underestimated by the fact that hospitalization was mandatory until June 2020 for all patients with SARS-CoV-2 in Argentina, including those with mild or moderate disease. However, most of the enrollment in this study occurred when hospitalization was not mandatory for such cases. Second, the complete cohort enrolled was not analyzed in this report. Enrollment which has occurred at the end of the study period was under ongoing collection of data at the time of this analysis. Given the similarity of results in the current study with several others, it is probable that this partial analysis truly reflects the real universe of patients hospitalized with COVID-19 in Argentina. Last, variables associated with ICU admission or 30-day mortality were not validated in a complementary dataset of subjects. Despite these limitations we believe our study provides key clinical information to better understand the characteristics of patients hospitalized with COVID-19. The current investigation further validates in our population several observations made in patients hospitalized with COVID-19 in different world regions. Finally, using clinical and laboratory parameters available at bedside this study identifies factors associated with adverse clinical outcomes in patients hospitalized with COVID-19.

## Supporting information

S1 Data (XLSX)
